# Severe burn injuries and the impact of mental health: insights from 7 years at Switzerland’s leading burn center

**DOI:** 10.1007/s11739-025-03887-6

**Published:** 2025-02-12

**Authors:** Michael-Alexander Pais, Mauro Vasella, Oliver Matthes, Elena Millesi, Alexander Kobler, Tabea Breckwoldt, Gregory Reid, Lukas Naef, Luzie Hofmann, Jennifer Ashley Watson, Philipp Karl Bühler, Pietro Giovanoli, Bong-Sung Kim

**Affiliations:** 1 Department of Plastic Surgery and Hand Surgery, University Hospital Zurich, Zurich, Switzerland; 2 Department of Plastic and Reconstructive Surgery, BG Trauma Center Ludwigshafen, University of Heidelberg, Heidelberg, Germany; 3 Department of Consultation-Liaison Psychiatry and Psychosomatic Medicine, University Hospital Zurich, Zurich, Switzerland; 4 Division of Plastic, Reconstructive and Aesthetic Surgery, Medical University of Vienna, Vienna, Austria; 5 Department of Hand and Plastic Surgery, Kantonsspital Frauenfeld, Frauenfeld, Switzerland; 6 Center of Intensive Care Medicine, Kantonsspital Winterthur, Winterthur, Switzerland

**Keywords:** Burns, ICU, Substance abuse, Psychiatric predisposition

## Abstract

Severe burn injuries present significant global healthcare challenges, with outcomes significantly influenced by factors such as socioeconomic status, mental health conditions, and substance use, which vary across regions and healthcare systems. This study investigates the etiologies, treatment outcomes, and the impact of psychiatric conditions and controlled substance use on burn injuries in patients admitted to the intensive care unit at Switzerland’s largest Burn Center. Data were retrospectively analyzed from 438 patients admitted to the University Hospital Zurich Burn Center ICU between 2016 and 2022. Variables assessed included baseline characteristics, burn etiologies, injury mechanisms, treatment modalities, complications, discharge outcomes, and mortality. Statistical analyses employed generalized linear models and logistic regression. Most burns occurred at home/leisure activities (43.4%), 21.2% at work. Pre-existing psychiatric conditions were present in 38.8% of patients, and 24.4% were under influence of controlled substances at the time of injury. These factors were associated with higher complication rates, increased surgeries, longer hospital stays, and lower survival rates. The overall in-hospital mortality rate was 15.8%, significantly linked to a history of controlled substance use. These findings highlight the significant impact of psychiatric conditions and use of controlled substances on burn injury outcomes, underscoring the importance of an interdisciplinary approach to treatment and management, particularly for patients with mental health and substance abuse histories.

**Supplementary Information:**

The online version contains supplementary material available at 10.1007/s11739-025-03887-6.

## Background

Severe burn injuries represent a significant burden on healthcare systems worldwide, representing multifaceted challenges in management and recovery [1]. These injuries can arise from a myriad of causes, ranging from thermal sources such as flames, scalding liquids, or hot surfaces, which constitute the majority of etiologies [2, 3], to chemical exposure, electrical accidents, and radiation. Each etiology carries its unique set of complications, necessitating a nuanced understanding for effective treatment and prevention strategies [4].

According to the World Health Organization, burn injuries cause around 180′000 deaths worldwide with the majority occurring in low- to middle income countries [5]. Smolle et al*.* systematically reviewed the literature regarding burn epidemiology worldwide from 2001 to 2016 and noticed an overall decrease in incidence, severity, hospitalization time, and mortality, however, this was mostly noticeable in developed, high-income countries [2]. Data for medium-income countries, on the other hand, were sparse and for low-income countries even sparser. The authors of the study underlined the need for an international burn database.

Switzerland, the fifth wealthiest country in the world in 2022 in terms of GDP per capita and a GDP of 818.4 billion USD, has a population of 8.776 million, a high-level education system, and an excellent and expensive (ca.100 billion USD per year) general health care system [6]. According to the Swiss Council for Accident Prevention BFU, most burns occur within homes and during leisure activities (8000 patients per year), which is 70% of all cases per year according to the Swiss National Accident Insurance Fund (SUVA), and a fifth of all incidents occur at the workplace [7, 8].

Within low-, middle-, and high-income countries the risk for burns correlates with the socioeconomic status [9–12]. Yet, when looking into other risk factors leading to burn injuries, there seems to be discrepancies. Preexisting substance abuse [13–15] and psychiatric disorders have been described as a risk factor in burn injuries, a higher rate of complications, longer hospitalization, and a higher mortality, however, data regarding possible correlations with the outcome in burn injuries have been scarce [16, 17].

The objective of this study is to provide a comprehensive overview of the etiologies and related factors surrounding thermal injuries among intensive care unit (ICU) patients receiving treatment at Switzerland’s largest Burn Center for adults. Our investigation aims to explore associated sociodemographic factors, diverse causes or etiologies, injury mechanisms, burn-specific characteristics, treatment, complications, discharge, and mortality data. We aim to highlight the connection between burns and various factors, especially pre-existing psychiatric conditions or prior use of controlled substances, such as antipsychotic medication.

## Materials and methods

This retrospective study was approved by the cantonal ethical committee of Zurich, Switzerland and registered in accordance with the Declaration of Helsinki in the “Registry of all Projects in Switzerland” of Swiss Ethics Committees on research involving humans (BASEC 2017-01681). General consent (GC) and informed consent was obtained from all subjects and/or their legal guardian(s) for data collection. Data from patients admitted consecutively between 2016 and 2022 to the ICU of the Burn Center at the Department of Plastic Surgery and Hand Surgery, University Hospital Zurich, were analyzed. Exclusion criteria were a rejected GC and patients under 16 years of age. All analyses were based on data available at the time of hospital admission and hospitalization. Information on comorbidities were obtained from the patients, their general practitioner’s records, and available medical histories.

Frequency distribution tables were used to summarize data on baseline characteristics, such as demographic information, various etiologies of burns, injury mechanisms, burn-specific characteristics, treatment, complications, discharge, and mortality data. Generalized linear models were employed for count, and logistic regression analyses for categorical data, respectively, to analyze the association of these characteristics with predefined outcome measures.

### Baseline characteristics and demographic data

Collected patient-related data included: age, gender, obesity (defined as a body mass index (BMI) ≥ 30 kg/m [2]), diabetes mellitus, peripheral artery disease, coronary heart disease, heart insufficiency, chronic obstructive pulmonary disease (COPD), medical history of regular alcohol or nicotine consumption, pre-existing psychiatric conditions, multiple psychiatric preconditions (≥ 2), previous suicide attempts, use of controlled or illicit substances, and history of unemployment or retirement.

Pre-existing psychiatric conditions were based on the ICD-10 or the Diagnostic and Statistical Manual of Mental Disorders, 5th edition [18], including conditions F06, 07, 09, 20–41, 43, 44, 60–63, 68, 69, 91–94, 98, 99.

Controlled substances included general antipsychotics, tricyclic antidepressants, sedatives or combinations. Illicit substances investigated included tetrahydrocannabinol, methadone, buprenorphine, other cannabinoid and combinations of these substances, as well as conditions listed in the ICD-10 under F11–16, 18, 19 and 55 (nicotine and alcohol listed separately above).

Further data included analysis weekday and month of admission.

### Etiology of burn injuries

Burns were classified based on their potential causes, distinguishing between those that happened at home, occurred during leisure activities and those being work-related incidents or traffic accidents. Within the scope of recurrent etiologies for home injuries were fuel-/petroleum-related incidents, explosion-related cases, barbecue-related incidents, cooking-related accidents, and house-fire-related burns. Additional distinction was made between burn injuries directly linked to alcohol- or nicotine-consumption.

Injuries associated with existing psychiatric conditions, including injuries resulting from suicide attempts were also highlighted. Furthermore, injuries related to neurological conditions such as cerebral ischemia, hemorrhage, multiple sclerosis, paraplegia, epilepsy, dementia, and medical conditions such as syncope, were included. Finally, exposure to uncommon causes of injury such as train surfing, lightning strikes, and sauna visits were assigned to the category “extreme”.

### Analysis of injury mechanisms, burn-specific characteristics, treatment, complications, discharge and mortality data

The data analysis incorporated burn injury mechanisms and specific characteristics, in addition to treatment modalities, complications, discharge data and mortality.

Burn injuries were classified according to their injury mechanism, *i.e.* scalding, explosion, flame exposure, electrical burns, or chemical burns. Frostbite injuries were included as a separate category.

Burn-specific characteristics analyzed included the abbreviated burn severity index (ABSI) and revised Baux scores, burns exceeding > 20% total body surface area (TBSA), those surpassing > 10% TBSA in individuals over 65 years of age, burns affecting the face, hands, genitals, and larger joints, as well as deep-partial thickness (DPTB), full-thickness burns (FTB), circular burns, verified inhalation injury (IHI), and additional trauma (e.g., ocular injuries, traumatic brain injuries, or middle facial fractures).

Treatment-specific data included the continuous counts of the number of surgeries, cultured epithelial autografts/keratinocyte (CEA) and enzymatic debridement with Nexobrid® (MediWound, Yavne, Israel).

Complications taken into account were wound infections, loss of skin grafts or transplants, respiratory infections, urinary tract infections, bacteremia, sepsis, multiorgan failure, gastroparesis, refeeding syndromes, gastrointestinal bleeding, ileus, renal insufficiency, electrolyte disorders, rhabdomyolysis, anemia, vasoplegia, thrombosis, embolisms, cardiac failure (including arrhythmias), agitation, delirium, post-traumatic stress disorder, and post-traumatic depression.

The discharge data included discharges to home, rehabilitation facilities, or other institutions that included home-based burn units for repatriation, regionalization facilities, nursing homes, and psychiatric care facilities.

Moreover, the duration of overall hospitalization and in-hospital mortality were also included in the analyses.

### Statistical analyses

Frequency distribution tables were employed for descriptive statistics of data discussed in the preceding chapters.

Predetermined primary outcome included the frequency of surgeries, incidence of complications, duration of hospitalization. These were treated as count data and analyzed using a generalized linear model (GLM) with a negative binomial distribution to account for overdispersed distributions. The models controlled for confounding variables, namely patients’ baseline characteristics, demographic data, burn-specific factors, and treatment-related variables. This approach also addressed the potential influence of confounders, such as “psychiatric condition” and “substance abuse”.

Incident rate ratios (IRR) were estimated for: patient (age, pre-existing psychiatric condition, previous use of controlled or illicit substances, injury related to alcohol consumption, and status of employment), injury (ABSI score, > 20% TBSA, burns of the face, hands, genitals, and larger joints, and presence of verified IHI), treatment (recurrent surgeries ≥ 2, CEA and Nexobrid®), and complication-specific data (complications ≥ 3, wound infection), as well as information regarding admissions to rehabilitation.

Survival rate was considered as a categorical variable and analyzed using logistic regression for binary data. Estimates represented the log odds of “survival = yes” versus “survival = no”. The survival rate was also estimated for relevant patient (age, pre-existing psychiatric condition, previous use of controlled or illicit substances, injury related to alcohol consumption, and status of employment), injury data (ABSI score, > 20% TBSA, burns of the face, hands, genitals, and larger joints, and presence of verified IHI), recurrent surgeries ≥ 2, complications ≥ 3, and admissions to rehabilitation.

Sensitivity analyses were conducted to evaluate the robustness of the results. These analyses involved:

- Exclusion of outliers: Patients with extreme values (e.g., TBSA > 80%, ABSI > 10) were excluded to test whether these individuals disproportionately influenced the findings.
- Alternative: Continuous predictors (e.g., age, TBSA) were categorized into clinically meaningful groups to ensure consistency of results across different modeling approaches.
- Handling of missing data: Analyses were repeated after excluding cases with missing data for psychiatric conditions or substance use.

Across all sensitivity analyses, the observed associations and effect estimates remained consistent, confirming the robustness of the results.

Statistical analyses were conducted using Jamovi statistics software (v2.3, The Jamovi project, Sydney, Australia). Continuous count variables were represented as mean ± standard deviation (SD), patient ages as median (IQR), and categorical variables as counts and %.

GraphPad Prism (v9.5.1, GraphPad Software, San Diego, California, USA), was also used for statistical analyses, with values presented as mean ± standard error of mean. Data distributions were assessed using the D’Agostino-Pearson and Shapiro–Wilk’s tests. For non-normally distributed data, the Kruskal–Wallis test, with Dunn’s post hoc analysis for multiple comparisons, was employed for between-group analyses. Statistical significance was considered for *p*-values < 0.05.

## Results

### Baseline characteristics and demographic data

A total of 438 patients admitted to the ICU were included in this study, with the majority being male (73.5%) and a median age of 42.0 (IQR: 15–93). The most common comorbidities upon admission were a medical history of alcohol (27.5%) and nicotine abuse (22.0%) previous to the injury. A fourth of all patients were retired (25.9%). Detailed baseline characteristics and comorbidities recorded upon admission can be found in Table 1.

**Table 1:** Baseline demographic data

Patient characteristics (*n* = 438)	Percentage (%), or median (IQR)
Gender Male Female	73.5 26.5
Age	42.0 (15–93)
BMI^a^ $\ge$ 30 kg/m^2^	13.7
Diabetes mellitus	6.9
Peripheral artery disease	11.3
Coronary heart disease	8.6
Heart insufficiency	4.2
COPD ^b^	4.7
Nicotine consumption	22.0
Alcohol consumption	27.5
Pre-existing psychiatric condition	38.8
Multiple psychiatric preconditions ($\ge$ 2)	10.1
Previous suicide attempts	10.6
Controlled substances	24.4
Unemployed	11.2
Retired	25.9

Data is given for Continuous variables in (%) or median (IQR), frequency tables

^a^*BMI* Body Mass Index, ^b^*COPD* Chronic Obstructive Pulmonary Disease

### Pre-existing psychiatric comorbidities and history of controlled substances prior to burn injuries

Investigation of pre-injury prevalence of pre-existing mental health disorders was 38.8%, and 10.1% were suffering from multiple psychiatric conditions. Additionally, upon further examination of the medical records of all 438 patients, 24.4% were under treatment with controlled substances, and 10.6% had documented previous suicide attempts (Table 1). Illicit substances were consumed by 9 patients (2%) prior to the injury.

### Regional distribution, day of admission, and seasonal variations in admissions

**Fig. 1:**
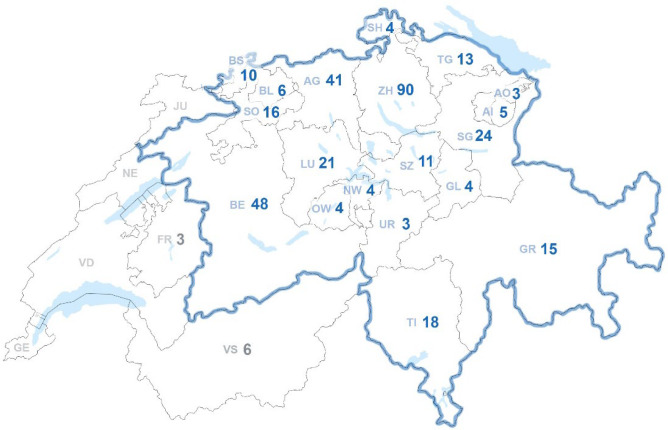
Absolute number of cantonal admissions for severe burn injuries. A total of 438 patients were admitted to the Burn Center at the University Hospital Zurich. The cantons of Zurich (ZH) and Berne (BE) accounted alone for almost a third of all cases

**Fig. 2:**
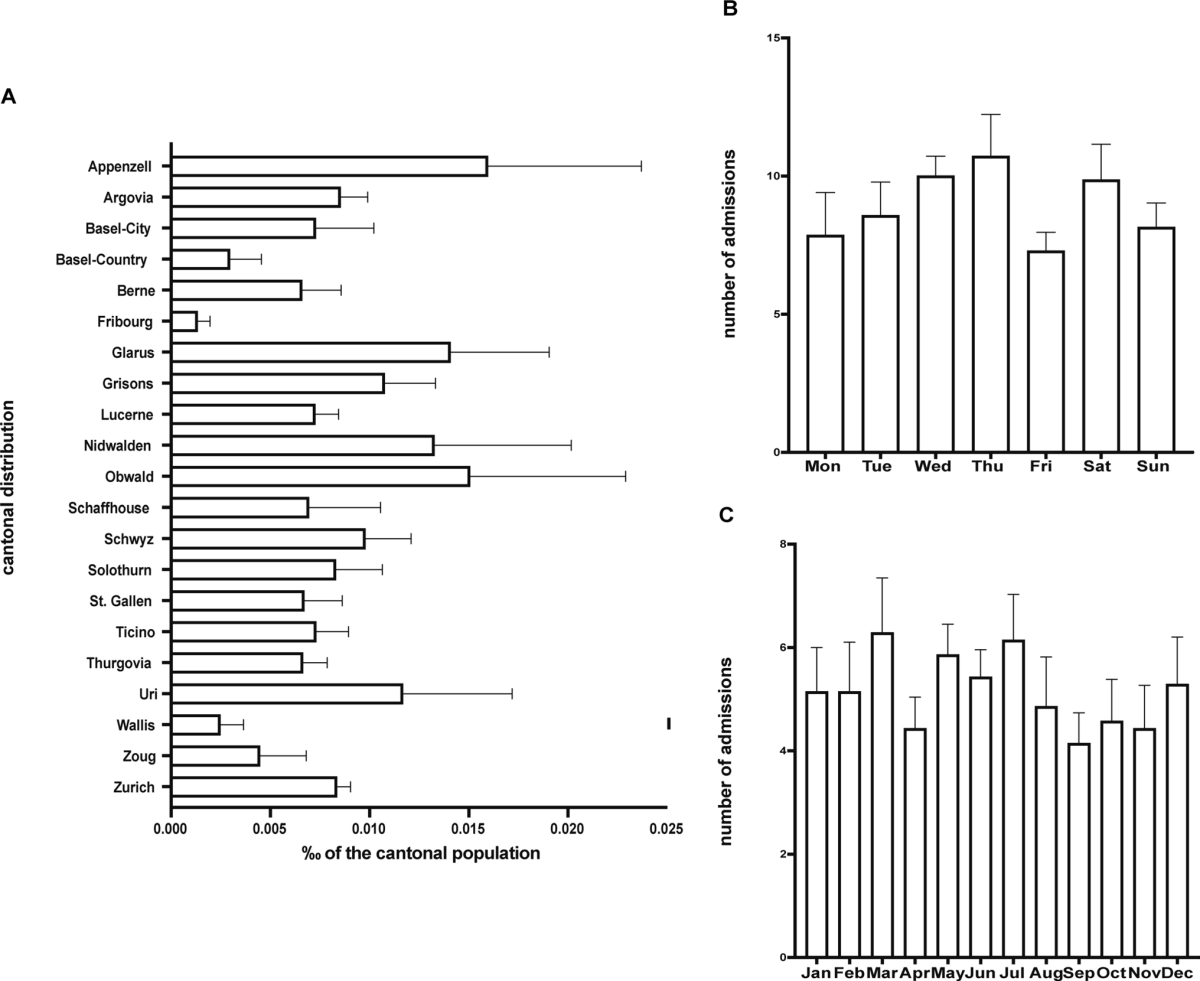
**A** Cantonal distribution of burn injuries per year. Descriptive count data of the number of admissions per canton, indicated as [‰] of the recorded cantonal population, as of 2019. Data = per thousand. Kruskal–Wallis analysis with Dunn`s post hoc for multiple comparisons indicated no significant differences between cantons. **B** Day-of-the-week admissions for burn injuries. Descriptive count data of binary variables. Data = counts and %. Kruskal–Wallis analysis with Dunn`s post-hoc for multiple comparisons indicated no significant differences between the days of the week. **C** Seasonal variation for burn-injury admissions**.** Descriptive count data of binary variables. Data = counts and %. Kruskal–Wallis analysis with Dunn`s post hoc for multiple comparisons indicated no significant differences between the different months

Analyzing the absolute number of cantonal admissions for burn injuries per year in relation to the population count and indicated as [‰], revealed that the highest number of admissions for burn injuries occurred in the rural half-cantons (half cantons are small full cantons that have split in history) of Appenzell Inner Rhodes and Outer Rhodes, while the fewest patients were recorded in the canton of Fribourg which usually refer their patients to the second burn center of Switzerland, the Lausanne University Hospital (Fig. 1). Notably, no patients were reported from Geneva, Jura, Neuchatel, and Vaud, given that they are also mainly admitted to Lausanne (Fig. 2A).

When assessing the frequency of burn injury admissions throughout the week, Thursdays showed the highest rate of patient admissions due to severe burn injuries (Fig. 2B). When looking at the distribution across different months of the year based on burn injury admissions, the majority of patients were admitted in March (Fig. 2C.).

Analysis for multiple comparisons indicated no significant differences between cantons, seasons, or days of the week.

### Etiology of burn injuries

The largest proportion of burn injuries occurred at home or during leisure activities (43.4%). The most common causes associated with home activities were cooking-related incidents (10.5%), followed by house fires (9.8%). Among burn injuries, 24.9% were associated with a current or acute psychiatric event and 14.8% were self-inflicted burns or related to acute suicide attempts. Additionally, a high portion of injuries stemmed from work-related incidents (21.2%), whereas only 2.3% were due to traffic accidents. Neurological or medical events leading to the injury, such as epileptic seizures or syncope, accounted for 13.3% of burn injuries. Nicotine consumption was a documented factor contributing to burn injuries in 14.4% of the cases. Alcohol-consumption-related burns constituted 5.5% of the cases. Furthermore, special injury conditions related to train surfing, lightning incidents, and sauna visits were responsible for 2.1%, 0.5%, and 1.6% of the burn injuries, respectively. Detailed information regarding the causes of injury is depicted in Table 2.

**Table 2:** Injury-specific characteristic based on the potential causes of burns

Group or cause-specific characteristics (*n* = 438)	
	Percentage (%)
Any home or leisure injuries	43.4
Fuel-, petroleum-related	7.5
Explosion-related	7.8
Barbecue-related	6.4
Cooking-related burns	10.5
House-fire-related burns	9.8
Alcohol-consumption-related	5.5
Nicotine-consumption-related	14.4
Current-psychiatric-condition-related	24.9
Injury related to suicide attempts	14.8
Neurologic/ medical condition	13.3
Work-related burns	21.2
Traffic-associated burns	2.3
Injury related to extreme conditions	2.3
Train surfing	2.1
Lightning	0.5
Sauna	1.6

Binary variables, frequency tables

#### Injury mechanisms and other burn-specific characteristics

For burn mechanisms, the largest proportion of injuries resulted from flame-related incidents (64.2%), followed by scalding injuries (51.1%), electric burns (11.6%), chemical burns (3.7%), and others (5.4%; e.g., injuries related to frostbite).

**Fig. 3:**
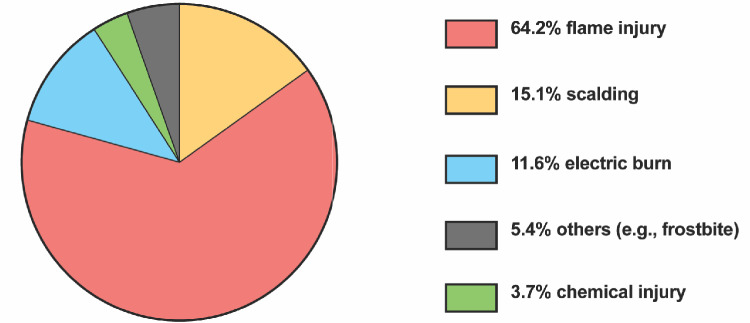
Burn-injury-specific mechanisms. Descriptive count data of binary variables relating to mechanisms for burn injuries. Data = counts and %

The mean ABSI score was 6.9 ± 2.63), and mean Baux score was 74.7 ± 28.3. In 51.8% of the cases, a TBSA greater than 20% was recorded. Among the patients admitted to the ICU, 20.1% had involvement of the face, hands, genitals, and joints, while 82.2% exhibited DPTB and full-thickness burns FTB. Additionally, 36.5% had circular burns. IHI was present in 30% of cases, and 25.3% experienced additional trauma. Figure 3 and Table 3 illustrate the burn injury-related characteristics.

**Table 3:** Burn-specific characteristics (*n* = 438)

Mechanism of injury	Percentage (%) or mean ± SD
Scalding	15.1
By flame	64.2
Electric burn	11.6
Chemical burn	3.7
Others (e.g., frostbite)	5.4
ABSI score ^a^	6.9 (2.63)
Baux score	74.7 (28.3)
> 20% TBSA ^b^	51.8
> 10% TBSA ^b^, 65 years of age	20.1
Burns of the face, hands, genitals, and larger joints:	82.2
Deep-partial and full-thickness burns	71.5
Circular burns	36.5
Verified IHI ^c^	30.0
Additional trauma	25.3

Continuous variables ± SD, binary variables respectively, frequency tables

^a^*ABSI* Abbreviated Burn Severity Index, ^b^*TBSA* Total Body Surface Area, ^c^*IHI* Inhalation injury

#### Therapeutic modalities, complications, hospitalization, discharge and mortality rates

On average, patients underwent 3.56 surgeries ± 3.6, with 10% receiving CEA treatment and 28.8% being debrided enzymatically with Nexobrid®. The mean complication rate per patient was 2.93 ± 3.27, with 23.6% experiencing wound infections, 7.1% experiencing losses of skin grafts, and 18.7% being diagnosed with post-traumatic mental health issues, such as post-traumatic depression. Mean hospitalization was 30.7 days ± 43.3. Upon discharge, the largest proportion were transferred to rehabilitation facilities (35.8%), while 31.1% returned home, both initially transitioning through the regular plastic surgery ward. Additionally, 14.8% were discharged to other institutions, including repatriation of patients to a Burn Center of their respective home countries, regionalization to local hospitals of the patients’ residence, nursing homes, and psychiatric care facilities. The in-hospital mortality rate was 15.8% (*n* = 69) of which 36 patients (52.2%) received palliative care/comfort therapy which was agreed upon by a multidisciplinary team of physicians, assistance of the medical ethics team, and the patients/legal representative in case no pre-defined patient decree was available. The results are summarized in Supplemental Table 1.

#### Association between the number of surgeries and a history of pre-existing psychiatric conditions

Our initial assessment of outcome measures examined possible correlations between the number of surgeries and various risk factors (Supplemental Table 2).

ignificant interactions were identified between pre-existing psychiatric conditions and IHI (*p* = 0.014, IRR: 1.528), suggesting a higher risk for recurrent surgeries among individuals with a history of pre-existing psychiatric conditions in association with IHI. Moreover, a significant correlation between an increased number of surgeries and a history of alcohol consumption (*p* = 0.002, IRR: 0.765), ABSI scores, and burns covering more than 20% TBSA was observed (*p* < 0.001, IRR: 1.063; and *p* = 0.034, IRR: 1.227, respectively).

#### Association between complication rate and a history of pre-existing psychiatric conditions and positive history of controlled substances

Our findings revealed a significant positive correlation between the complication rate and pre-existing psychiatric conditions. Additionally, we noted a similar correlation with a history of controlled substance usage and complication rates (*p* = 0.046, IRR: 0.806, and *p* = 0.004, IRR: 1.411, respectively). This suggests a risk for complications among individuals with a history of pre-existing psychiatric conditions, while indicating an even higher risk with for those with a history of controlled substances.

Additionally, significant interactions were identified between pre-existing psychiatric conditions and ABSI scores (*p* = 0.017), as highlighted in Supplemental Table 3.

#### Association between length of hospitalization and a history of pre-existing psychiatric conditions

The analysis revealed noteworthy interactions between pre-existing psychiatric conditions and IHI (*p* = 0.03, IRR: 1.190), suggesting a higher risk for increased hospitalization time among individuals with a history of pre-existing psychiatric conditions in association with IHI.

In addition, a significant correlation between the duration of hospitalization and age at admission was noted (*p* = 0.031). The results are highlighted in Supplemental Table 4.

#### Association between survival rate and a history of controlled substances

History of controlled substances showed statistical significance (*p* = 0.036, OR: 0.354, 95%-CI 0.134 to 0.936), highlighting that survival was less likely among these patients.

A detailed illustration can be found in the Supplemental Table 5.

## Discussion

The ICU cohort predominantly comprised male patients with elevated ABSI scores, TBSA exceeding 20%, and significantly increased risks for multiple surgeries, complications, prolonged hospital stays, and decreased survival rates, consistent with existing literature [19, 20]. Most patients sustained injuries at home or during leisure activities (43.4%), which is notably lower than the 71% reported by the Swiss Council for Accident Prevention, likely due to the inclusion of non-ICU patients in their data [7]. Work-related accidents accounted for approximately 21.2%, congruent with official data, reflecting ongoing occupational risks despite improved safety measures [21, 22]. A significant proportion of injuries occurred in retired individuals, consistent with the demographic composition of Switzerland [23].

Burn etiologies were primarily domestic, with cooking-related incidents (10.5%) and house fires (9.8%) being the most frequent causes. Notably, 24.9% of burns were linked to acute psychiatric events, and 14.8% were self-inflicted or related to suicide attempts, highlighting the vulnerability of individuals with mental health conditions to severe burn injuries [17, 24]. Neurological or medical events leading to burn injuries, including seizures and syncope, were also significant contributors (13.3%), while substance abuse, particularly nicotine (14.4%) and alcohol (5.5%), played critical roles. Alcohol-related burns are often associated with more extensive injuries, possibly due to cognitive and coordination impairments [14, 15]. A meta-analysis of 17 studies, by Klifto et al*.*, demonstrated that smoking was associated with a higher rate of intubation and infection, while alcohol correlated with increased intubation, inhalation injury, ventilator days, ICU stays, and mortality [13].

Geographically, admissions were highest in the rural half-cantons of Appenzell, with the lowest in Fribourg, which refers patients to Lausanne. This pattern aligns with literature suggesting that rural patients often experience more severe and extensive injuries [25, 26]. The highest admission rates occurred on Thursdays, with most patients admitted in March; however, statistical analysis indicated no significant differences in admissions across cantons, seasons, or weekdays, and no significant outcome differences. Schiefer et al*.* analyzed 25 years of data from their burn center in Germany, observing the highest rate of admissions on Saturdays; nevertheless, these data were only correlated with median admission time and showed no statistical significance [27]. An investigation by Taira et al*.,* focusing on “off-hours” admissions, without naming specific weekdays, of burn patients and its possible impact on outcome, demonstrated no statistical difference in mortality rates between the two groups [28]. However, none of the aforementioned studies analyzed data seasonal admission rates.

The overall mortality rate observed in this study was 15.8%, with an 8.1% mortality rate among patients with burns exceeding 20% TBSA. Most lethal outcomes were due to palliative or comfort care decisions (52.2%), often influenced by legal directives and family discussions. Swiss patients frequently have advanced directives that influence treatment decisions, even when medical intervention might be possible. There are no official numbers, however, there is an increasing trend of patients with a written provision or will admitted at the Burn Center in Zurich. Cases without such directives were discussed by multidisciplinary teams with support from medical ethics experts.

The Swiss mortality rate for severely burned ICU patients (7.5%) is significantly lower than rates reported by the American Burn Association (17.8%) and in Ethiopia, as examples of high- and low-income countries, highlighting disparities in burn care and outcomes internationally [29, 30]. Comparative data from Norway, a country with similar healthcare standards, reported an overall mortality rate of 4.4%, which increased to 20% with burns over 20% TBSA. Contributing factors may include limited access to burn centers and challenging geography, often necessitating a medevac by helicopter which can be delayed by rapidly changing weather conditions, emphasizing the critical impact of timely access to specialized care [31]. Although transportation delays up to 16 h do not significantly affect outcomes, longer delays remain poorly studied and may exacerbate mortality rates [32–34].

Despite having access to excellent healthcare infrastructures, mental health disorders are common among the Swiss population [35]. A significant portion of our cohort presented with pre-existing mental health conditions, substance abuse, or acute intoxication at the time of injury. Notably, 38.8% of patients had a psychiatric disorder, and 24.9% were experiencing acute psychiatric crises leading to injury. These findings are consistent with prior research linking 28–75% of burn injuries to psychiatric conditions, underscoring the need for integrated mental health support in burn care [17, 24, 36, 37]. Won et al*.* found that 6% of burn patients had psychiatric or substance abuse comorbidities and were predominantly homeless males [17]. In comparison, reported rates of psychiatric disorders among burn patients in developing countries, such as Ethiopia, are significantly lower, likely due to underreporting and cultural stigmatization of mental health issues [38].

While psychiatric conditions did not correlate with extended hospital stays, increased surgery frequency, or elevated mortality in our study, they were associated with higher complication rates, consistent with previous research [17, 39, 40]. Psychiatric conditions, particularly those involving cognitive or emotional dysregulation, may impair patients’ ability to adhere to safety protocols, potentially contributing to burn injuries [41]. Additionally, antipsychotic medication can affect physiological factors such as metabolism and immune function, potentially influencing wound healing and increasing the risk of complications [42]. Use of controlled substances also correlated with increased complication risks and lower survival rates. These findings highlight the importance of integrating psychiatric care into burn management protocols. Routine psychiatric evaluations could help identify patients at higher risk for complications, enabling tailored interventions that address both psychological and physiological needs.

Patients undergoing multiple surgeries or requiring CEA demonstrated increased risks for complications and longer hospitalizations, likely due to the severity of their injuries. The correlation between surgery frequency and wound complications is in agreement with existing studies, emphasizing the challenges in managing complex burn wounds [24, 43, 44]. Interestingly, patients treated with enzymatic debridement exhibited reduced hospitalization and complication rates, although these findings may reflect selective application criteria rather than a direct treatment effect.

Overall, the study’s single-center data limit the generalizability of findings, particularly regarding socioeconomic factors, which were not directly analyzed. Moreover, this study is only representative of the German- and Italian-speaking part of Switzerland and patients ≥ 16 years of age, as the French-speaking part and patients < 16 years are covered by other institutions. However, the considerable cohort size provides valuable insights into burn injury management in Switzerland.

## Conclusion

Our study illustrates a direct link between certain outcomes—such as the number of surgeries, complication rates, hospitalization duration, and survival rates—and pre-existing psychiatric conditions, prior use of controlled substances, or alcohol consumption. This highlights the vulnerability of people suffering from mental health issues and substance abuse when it comes to burn injuries, and the need of education regarding prevention. Additionally, these findings emphasize importance of comprehensive, multidisciplinary approaches in managing burn patients, particularly those with complex psychiatric or substance abuse histories, to optimize outcomes and reduce the burden of burn injuries on healthcare systems.

## Supplementary Information

Below is the link to the electronic supplementary material.

Supplementary file1 (DOCX 15 KB)

Supplementary file2 (DOCX 16 KB)

Supplementary file3 (DOCX 17 KB)

Supplementary file4 (DOCX 16 KB)

Supplementary file5 (DOCX 16 KB)

## Data Availability

The datasets used and/or analyzed during the current study are available from the corresponding author on reasonable request.

## Abbreviations

ABSI: Abbreviated burn severity index
CEA: Cultured epithelial autografts / keratinocyte
DPTB: Deep-partial thickness
FTB: Full-thickness burns
GC: General consent
GLM: Generalized linear model
COPD: Chronic obstructive pulmonary disease
ICU: Intensive care unit
IRR: Incident rate ratios
IH: Inhalation injury
SD: Standard deviation
TBSA: Total body surface area
