# Supplementary material for: Severe burn injuries and the impact of mental health: insights from 7 years at Switzerland’s leading burn center
Source: Intern Emerg Med. 2025 Feb 12;20(4):1141–51. doi: 10.1007/s11739-025-03887-6 (PMC12130154; doi:10.1007/s11739-025-03887-6)
Supplement: Supplementary file 1 — Supplementary file1 (DOCX 15 KB) [file 11739_2025_3887_MOESM1_ESM.docx]

**Supplemental Table 1. Therapy-specific characteristics (n = 438).**

|  | Percentage (%) or mean ± SD |
| --- | --- |
| Number of surgeries | 3.56 (3.6) |
| CEA ^a^ | 10.0 |
| Nexobrid^®^ | 28.8 |
| Complications | 2.93 (3.27) |
| Wound infections | 23.6 |
| Loss of transplant | 7.1 |
| Post-traumatic depression | 18.7 |
| Overall hospitalization time | 30.7 (43.4) |
| Discharge at home | 31.13 |
| Rehabilitation | 35.8 |
| Discharge, others | 14.8 |
| In-hospital mortality | 15.8 |

Continuous variables ± SD, binary variables respectively, frequency tables.

^a^ CEA = Cultured epithelial autograft.
