# Supplementary material for: Severe burn injuries and the impact of mental health: insights from 7 years at Switzerland’s leading burn center
Source: Intern Emerg Med. 2025 Feb 12;20(4):1141–51. doi: 10.1007/s11739-025-03887-6 (PMC12130154; doi:10.1007/s11739-025-03887-6)
Supplement: Supplementary file 2 — Supplementary file2 (DOCX 16 KB) [file 11739_2025_3887_MOESM2_ESM.docx]

**Supplemental Table 2. Regression results: number of surgeries and different risk factors.**

| Outcome: number of surgeries (counts), generalized linear model with negative binomial distribution | | |
| --- | --- | --- |
|  | **IRR ^a^ (95%-CI ^b^)** | **p-value** |
| **Age** | 0.998 (0.994 to 1.002) | 0.237 |
| **Pre-existing psychiatric condition** | 1.021 (0.859 to 1.215) | 0.815 |
| **Controlled substances** | 1.111 (0.930 to 1.327) | 0.248 |
| **Injury related to alcohol consumption** | 0.765 (0.645 to 0.906) | **0.002** |
| **Unemployed** | 1.016 (0.805 to 1.281) | 0.891 |
| **ABSI ^c^ score** | 1.063 (1.025 to 1.102) | **< .001** |
| **>20% TBSA ^d^** | 1.227 (1.012 to 1.488) | **0.034** |
| **Burns of the face, hands, genitals, and larger joints** | 0.937 (0.763 to 1.152) | 0.530 |
| **IHI ^e^ verified** | 0.890 (0.742 to 1.067) | 0.210 |
| **Complications** $\boldsymbol{\geq}$**3** | 1.587 (1.316 to 1.913) | **< .001** |
| **Wound infection** | 1.418 (1.186 to 1.694) | **< .001** |
| **Rehabilitation** | 1.263 (1.077 to 1.482) | **0.004** |
| **Pre-existing psychiatric condition “and” IHI** ^d^ | 1.528 (1.090 to 2.146) | **0.014** |

Relevant association between the number of surgeries and history of alcohol consumption,

Abbreviated Burn Severity Index score, >20% Total Body Surface Area, wound infections, complication rate $\geq$3, and admission to rehabilitation, respectively. GLM regression model with negative binomial distribution and a log link due to a skewed distribution. Significant interactions between psychiatric condition, pre-existing, and inhalation injury.

^a^ IRR = Incidence Rate Ratio, ^b^ CI = Confidence Interval, ^c^ ABSI = Abbreviated Burn Severity Index, ^d^ TBSA = Total Body Surface Area, ^e^ IHI = Inhalation injury.
