# Supplementary material for: Severe burn injuries and the impact of mental health: insights from 7 years at Switzerland’s leading burn center
Source: Intern Emerg Med. 2025 Feb 12;20(4):1141–51. doi: 10.1007/s11739-025-03887-6 (PMC12130154; doi:10.1007/s11739-025-03887-6)
Supplement: Supplementary file 3 — Supplementary file3 (DOCX 17 KB) [file 11739_2025_3887_MOESM3_ESM.docx]

**Supplemental Table 3. Regression results: risk factors for different complications.**

| Outcome: complications (counts), generalized linear model with negative binomial distribution | | |
| --- | --- | --- |
|  | **IRR ^a^ (95%-CI ^b^)** | **p-value** |
| **Age** | 0.999 (0.994 to 1.004) | 0.681 |
| **Pre-existing psychiatric condition** | 0.806 (0.652 to 1.85) | **0.046** |
| **Controlled substances** | 1.411 (1.117 to 1.786) | **0.004** |
| **Injury related to alcohol consumption** | 0.947 (0.757 to 1.186) | 0.634 |
| **Unemployed** | 1.083 (0.795 to 1.480) | 0.611 |
| **ABSI score ^c^** | 1.123 (1.065 to 1.186) | **< .001** |
| **>20% TBSA ^d^** | 1.343 (1.038 to 1.738) | **0.022** |
| **Burns of the face, hands, genitals, and larger joints** | 1.241 (0.933 to 1.653) | 0.143 |
| **IHI ^e^ verified** | 1.074 (0.851 to 1.354) | 0.533 |
| **Number of surgeries** $\boldsymbol{\geq}$**2** | 1.444 (1.065 to 1.964) | **0.018** |
| **CEA ^f^** | 1.790 (1.325 to 2.433) | **< .001** |
| **Nexobrid^®^** | 1.023 (0.821 to 1.275) | 0.838 |
| **Rehabilitation** | 1.455 (1.174 to 1.804) | **< .001** |
| **Pre-existing psychiatric condition “and” ABSI ^c^ score** | 1.093 (1.008 to 1.185) | **0.017** |

Clear significant association between complications and pre-existing psychiatric conditions, controlled substances, Abbreviated Burn Severity Index score, >20% Total Body Surface Area, number of surgeries $\geq$2, CEAs, and admissions to rehabilitation, respectively. GLM regression model with negative binomial distribution and a log link due to a skewed distribution. Significant interactions between psychiatric condition, pre-existing, and ABSI ^c^ score.

^a^ IRR = Incidence Rate Ratio, ^b^ CI = Confidence Interval,

, ^c^ ABSI = Abbreviated Burn Severity Index, ^d^ TBSA = Total Body Surface Area, ^e^ IHI = Inhalation injury, ^f^ Cultured epithelial autograft (CEA).
