# Supplementary material for: Severe burn injuries and the impact of mental health: insights from 7 years at Switzerland’s leading burn center
Source: Intern Emerg Med. 2025 Feb 12;20(4):1141–51. doi: 10.1007/s11739-025-03887-6 (PMC12130154; doi:10.1007/s11739-025-03887-6)
Supplement: Supplementary file 4 — Supplementary file4 (DOCX 16 KB) [file 11739_2025_3887_MOESM4_ESM.docx]

**Supplemental Table 4. Regression results: hospitalization with different risk factors.**

| Outcome: hospitalization duration (days, counts), generalized linear model with negative binomial distribution | | |
| --- | --- | --- |
|  | **IRR ^a^ (95%-CI ^b^)** | **p-value** |
| **Age** | 0.995 (0.990 to 1.00) | **0.031** |
| **Pre-existing psychiatric condition** | 1.187 (0.970 to 1.451) | 0.083 |
| **Controlled substances** | 1.122 (0.920 to 1.374) | 0.254 |
| **Injury related to alcohol consumption** | 1.042 (0.874 to 1.246) | 0.645 |
| **Unemployed** | 1.058 (0.820 to 1.378) | 0.667 |
| **ABSI score ^c^** | 1.174 (1.106 to 1.247) | **< .001** |
| **> 20% TBSA ^d^** | 1.164 (0.937 to 1.448) | 0.153 |
| **Burns of the face, hands, genitals, and larger joints** | 1.011 (0.815 to 1.249) | 0.916 |
| **IHI ^e^ verified** | 1.221 (1.001 to 1.487) | 0.057 |
| **Number of surgeries** $\boldsymbol{\geq}$**2** | 2.045 (1.615 to 2.575) | **< .001** |
| **Nexobrid^®^** | 0.817 (0.687 to 0.974) | **0.021** |
| **Complications** $\boldsymbol{\geq}$**3** | 1.417 (1.153 to 1.744) | **< .001** |
| **Wound infection** | 1.137 (0.931 to 1.392) | 0.210 |
| **Rehabilitation** | 1.059 (0.882 to 1.273) | 0.535 |
| **Pre-existing psychiatric condition “and” IHI** ^e^ | 1.747 (1.190 to 2.557) | **0 .003** |

A positive association was found between hospitalization time and ABSI score, recurrent surgeries $\geq$ 2, Nexobrid therapy, complications $\boldsymbol{\geq}$ 3. GLM regression model with negative binomial distribution and a log link due to a skewed distribution. Patients with in-hospital mortality were not included in the model. Significant interactions between pre-existing psychiatric conditions, and IHI. ^a^ IRR = Incidence Rate Ratio, ^b^ CI = Confidence Interval, ^c^ ABSI = Abbreviated Burn Severity Index, ^d^ TBSA = Total Body Surface Area, ^e^ IHI = Inhalation injury.
