# Supplementary material for: Severe burn injuries and the impact of mental health: insights from 7 years at Switzerland’s leading burn center
Source: Intern Emerg Med. 2025 Feb 12;20(4):1141–51. doi: 10.1007/s11739-025-03887-6 (PMC12130154; doi:10.1007/s11739-025-03887-6)
Supplement: Supplementary file 5 — Supplementary file5 (DOCX 16 KB) [file 11739_2025_3887_MOESM5_ESM.docx]

**Supplemental Table 5. Regression results: survival rate with different risk factors.**

| Outcome: survival (%), logistic regression model | | |
| --- | --- | --- |
|  | **Odds ratio (95%-CI ^a^)** | **p-value** |
| **Age** | 0.991 (0.969 to 1.014) | 0.448 |
| **Pre-existing psychiatric condition** | 1.226 (0.469 to 3.208) | 0.248 |
| **Controlled substances** | 0.354 (0.134 to 0.936) | **0.036** |
| **Injury related to alcohol consumption** | 0.506 (0.199 to 1.289) | 0.153 |
| **Unemployed** | 1.415 (0.370 to 5.407) | 0.612 |
| **ABSI ^b^ score** | 0.600 (0.480 to 0.750) | **< .001** |
| **>20% TBSA ^c^** | 0.278 (0.090 to 0.880) | **0.029** |
| **Burns of the face, hands, genitals, and larger joints** | 1.883 (0.593 to 5.981) | 0.283 |
| **IHI ^d^ verified** | 0.377 (0.138 to 1.034) | 0.058 |
| **Number of surgeries** $\boldsymbol{\geq}$**2** | 3.368 (1.092 to 10.387) | **0.035** |
| **Complications** $\boldsymbol{\geq}$**3** | 0.394 (0.137 to 1.134) | 0.084 |
| **Rehabilitation** | 114.016 (16.457 to 789.925) | **< .001** |

Positive correlations were found between survival rate and controlled substances, ABSI score, >20 % TBSA, number of surgeries $\geq$ 2, and admission to rehabilitation. Logistic regression model for binary data. Estimates represent the log odds of "survival = yes" vs. "survival = no". No significant interactions.

^a^ CI = Confidence Interval, ^b^ ABSI = Abbreviated Burn Severity Index, ^c^ TBSA = Total Body Surface Area, ^d^ IHI = Inhalation injury.
